# Relationship between Mindfulness, Psychological Skills, and Mental Toughness in College Athletes

**DOI:** 10.3390/ijerph18136802

**Published:** 2021-06-24

**Authors:** Chih-Han Wu, Jui-Ti Nien, Chi-Yen Lin, Yu-Hsiang Nien, Garry Kuan, Tsung-Yi Wu, Fei-Fei Ren, Yu-Kai Chang

**Affiliations:** 1 Graduate Institute of Athletics and Coaching Science, National Taiwan Sport University, Taoyuan 333325, Taiwan; wu75757979@hotmail.com (C.-H.W.); iwwfy0224@gmail.com (J.-T.N.); 2 Physical Education Office, National Taiwan Ocean University, Keelung 202301, Taiwan; evalin@mail.ntou.edu.tw; 3 Department of Sport Performing Arts, University of Taipei, Taipei 111036, Taiwan; yhnien@utaipei.edu.tw; 4 Exercise and Sports Science Programme, School of Health Sciences, Universiti Sains Malaysia, Kelantan 16150, Malaysia; garry@usm.my; 5 Department of Combat Sport, National Taiwan University of Sport, Taichung 404401, Taiwan; 6 Department of Physical Education, National Taiwan Normal University, Taipei 106209, Taiwan; yukaichangnew@gmail.com; 7 Institute for Research Excellence in Learning Science, National Taiwan Normal University, Taipei 106209, Taiwan

**Keywords:** dispositional mindfulness, psychological skills, mental toughness, sports performance

## Abstract

Numerous studies have shown that dispositional mindfulness is positively associated with many mental abilities related to sports performance, including psychological skills and mental toughness. The purpose of this study was to explore the relationship between dispositional mindfulness, psychological skills, and mental toughness among different types of athletes. For this cross-sectional study, 101 college athletes were recruited. Their dispositional mindfulness, psychological skills, and mental toughness were measured by the Mindfulness Attention Awareness Scale (MAAS), Athletic Psychological Skills Inventory (APSI), and Traits of Mental Toughness Inventory for Sports Scale (TMTIS). Pearson’s correlation was used to calculate how dispositional mindfulness is associated with psychological skills and mental toughness. The results revealed that dispositional mindfulness is positively associated with comprehensive APSI (*r* = 0.21–0.36, *p* < 0.05), TMTIS overall (*r* = 0.27, *p* < 0.01), positive effort (*r* = 0.26, *p* = 0.01), and pressure (*r* = 0.30, *p* < 0.01). These findings suggest a positive linkage between mindfulness and the two examined psychological characteristics related to sports performance. Other approaches to increase mindfulness may be considered in the future.

## 1. Introduction

Mindfulness [1,2,3], psychological skills (e.g., motivation; attention, and confidence) [4], and mental toughness are considered to be psychological characteristics associated with optimal sports performance for elite athletes. These skills help the athletes establish effective coping strategies under pressure; enhance their performance in a sports context; and further distinguish successful athletes from less successful athletes [5]. Examining the relationship between these psychological characteristics is a crucial issue for developing the identification of talent and the improvement of performance by the athletes.

Mindfulness has been defined as paying attention to specific experiences in the present moment with awareness and without judgment [6]. It has also been described as the ability to change to an awareness, a focus, and an acceptance of the present experience [7]. Mindfulness can also be conceptualized as a dispositional trait (i.e., dispositional mindfulness), and it is considered one of the personality traits [4,8] that can be enhanced through regular training [9,10,11]. Several studies have demonstrated that better dispositional mindfulness is related to positive outcomes, such as higher psychological skills [12,13] and mental toughness [14,15], and to avoiding negative outcomes by having lower perceived stress [16,17], lessening burnout [18] and lowering competitive anxiety [9] in the athlete population. Moreover, better dispositional mindfulness has been found to be related to improvements in sports performance [10,11]. Taken together, existing research points to the value of examining mindfulness as one of the psychological characteristics of elite athletes.

These psychological skills are considered as a series of trainable psychological characteristics [4,11] that are required abilities when athletes have to deal with suddenly difficult situations that help them to improve their performance. These psychological skills also contribute to successful talent development and optimal performance by elite athletes [19] and include coping ability, motivation, and attention [20] or self-confidence and stress adjustment [21]. In addition, several studies have shown that successful athletes demonstrate more motivation [22], greater self-confidence [23], and concentration [24] than amateurs or sub-elite athletes.

On the other hand, mental toughness is generally referred to as an important psychological characteristic related to successful outcomes in elite sports [25] because research has found that its contributions to elite athletes are similar to those of psychological skills [26]. Mental toughness has been defined as the abilities of athletes to rebound from failure, cope with pressure, and face adversity [26]. Mentally tough athletes can still attain and sustain a higher performance state in different types of situations [27]. Several studies have shown that young athletes who report higher mental toughness are more likely to have lower anxiety [26] and more effective responses to dealing with stressful situations [28,29].

Previous studies have pointed out that dispositional mindfulness is positively related to psychological skills and mental toughness in athletes [13,14,15,17,30]. However, whether the relationship between these psychological characteristics among the multiple-sport population is consistent with previous studies that only investigated a single sport is still unknown due to the majority of the empirical studies focusing on a single sport instead of sports overall [14,15]. Identifying whether dispositional mindfulness links to psychological skills, and whether mental toughness is crucial for sports performance in multiple sports can confirm if these psychological characteristics are applicable among a variety of athletes. Therefore, the aim of the current study is to examine the correlation of dispositional mindfulness with psychological skills and mental toughness in multiple-sport populations. We hypothesized that higher dispositional mindfulness is more common among athletes who have higher psychological skills and mental toughness levels.

## 2. Methods

### 2.1. Participants

A total of 101 college athletes were recruited for this study. These participants, from National Taiwan Sports University, were college-level athletes from one of five sports: taekwondo, judo, archery, wushu, and tennis. The participants’ background characteristics and psychological status were measured by self-reporting, with the valid questionnaires being rated as 95% accurate. According to Declaration of Helsinki, this study was approved by the Institutional Review Board of Fu Jen Catholic University (C105152).

### 2.2. Questionnaire

#### 2.2.1. Chinese Version Mindful Attention Awareness Scale (CMAAS)

The dispositional mindfulness of athletes was measured by CMAAS [31], which is based on the Mindful Attention Awareness Scale (MAAS), a relatively stable measure and one of the most popular instruments for dispositional mindfulness [32]. The CMAAS is a 15 reverse-item, self-reporting measure, and each item rates as a 6-point Likert-type scale (1 = almost never to 6 = almost always), with a higher score indicating a high level of dispositional mindfulness. The internal consistency of the CMMAS is satisfactory (Cronbach’s alpha coefficients = 0.78).

#### 2.2.2. Athletic Psychological Skills Inventory (APSI)

The psychological skills of athletes were measure by APSI [33], which is based on the Athletic Coping Skills Inventory-28 (ACSI-28) [34]. APSI includes these five sub-scales: motivation, coachability, concentration, confidence, and peaking under pressure and coping with adversity. It contains 31 items, with each item rated on a 5-point Likert-type scale (1 = almost never to 5 = almost always). Higher scores indicate a higher athletic psychological skill. The internal consistency of overall, motivation, coachability, concentration, confidence, and peaking under pressure and coping with adversity are satisfactory (Cronbach alpha’s coefficients = 0.91, 0.92, 0.74, 0.73, 0.70, 0.85, respectively).

#### 2.2.3. Trait Mental Toughness Inventory for Sport (TMTIS)

Mental toughness of athletes was measured by TMTIS [35], which is currently one of the most frequently used measures of mental toughness in Taiwan. The instrument includes the three sub-scales: positive effort, antipressure, and endurance. The TMTIS contains 32 items, with each item rated on a 5-point Likert-type scale (1 = strongly disagree to 5 = strongly agree). Higher scores indicate a higher mental toughness. The internal consistency of overall, positive effort, antipressure, and endurance are satisfactory (Cronbach’s alpha coefficients = 0.95, 0.95, 0.87, 0.80, respectively).

### 2.3. Procedure

First, the coaches of each sports team were visited and informed about the research content, and permission for the data collection was obtained from them. Next, the participants received an explanation about how to fill in the questionnaire before their regular training. All participants are volunteers and all were notified about the anonymity of the study. they were allowed to withdraw at any time. Before filling out the questionnaire, participants provided their informed consent. Then the participants continued to fill in their demographic details, CMAAS, TMTIS, and APSI under the instruction and supervision of the research assistant. The total time was about 20 min.

### 2.4. Statistical Analysis

This study was analyzed utilizing SPSS 22.0 statistical software. Descriptive statistics are presented in the participants’ demographics. In addition, the Pearson’s correlation coefficients were conducted to assess the correlation between CMAAS score, APSI overall score, and APSI sub-scales scores. The analysis was also conducted for the correlation between CMAAS, TMTIS overall scores, and TMTIS sub-scales.

## 3. Results

### 3.1. Participants’ Demographic Characteristics and Levels of CMAAS, APSI, TMTIS

Table 1 summarizes the participants’ demographic characteristics, which contain age, educational level, and sport experiences, as well as their level of psychological characteristics including the CMAAS, APSI, and TMTIS. All descriptive data were presented by mean score and standard deviation.

### 3.2. Pearson Correlation Coefficients for CMAAS and APSI

Table 2 presents the results of the correlation between CMAAS score, APSI overall score, and APSI sub-scales scores.

There was a significantly positive correlation between the CMAAS and APSI overall score (*p* < 0.01) with a small correlation level (*r* = 0.36) (Cohen, 1992). There was also a significantly positive correlation between the CMAAS and APSI-sub-scales scores for motivation (*p* = 0.02), coachability (*p* = 0.02), concentration (*p* = 0.03), confidence (*p* = 0.001), peaking under pressure, coping with adversity (*p* = 0.03) with a small correlation level (*r* = 0.21–0.32) (Cohen, 1992).

Additionally, the correlation coefficient was statistically significant for the APSI overall score and five sub-scales (*p* < 0.01) with medium to large correlation levels (*r* = 0.52–0.81) (Cohen, 1992).

### 3.3. Pearson Correlation Coefficients for CMAAS and TMTIS

Table 3 presents the results of the correlation between CMAAS score, TMTIS overall score, and TMTIS sub-scales scores.

There was a significantly positive correlation between the CMAAS and TMTIS overall scores (*p* < 0.01) with a small correlation level (*r* = 0.27) (Cohen, 1992). There was also a significantly positive correlation between the CMAAS and TMTIS sub-scales scores for positive effort (*p* = 0.01) and anti-pressure (*p* < 0.01) with a small correlation level (*r* = 0.26–0.30) (Cohen, 1992). However, there was no significant correlation between the CMAAS and endurance (*p* = 0.24).

Additionally, the correlation coefficient was statistically significant at the TMTIS overall scores and three sub-scales (*p* < 0.01) with a large correlation level (*r* = 0.80–0.89) (Cohen, 1992).

## 4. Discussion

The study attempts to examine the relationship between dispositional mindfulness, psychological skills, and mental toughness in multiple-sport populations. Our primary findings revealed a positive correlation of dispositional mindfulness with the APSI overall measure and all domains of psychological skills. Additionally, a similar positive correlation was also observed between dispositional mindfulness and TMTIS overall measure, as well as with positive effort and anti-pressure of mental toughness.

Our findings are consistent with previous studies that observed the positive linkage between mindfulness and psychological skills [13,14,15,17,30]. The APSI used in this study has five sub-scales: motivation, coachability, concentration, confidence, and peaking under pressure and coping with adversity. These domains of psychological skill are considered to favor sports performance [26]. In terms of motivation, Ruffault, et al. [36] indicated that dispositional mindfulness is negatively correlated with external motivation and positively correlated with intrinsic motivation. That said, athletes with higher mindfulness status may focus better on their sport itself and derive more enjoyment, thereby reducing the possibility of dropping out of sports. Coachability refers to the attitude of athletes adapting and obeying their coach’s leadership and guidance and accepting the coach’s constructive criticism [37]. Jones and Hansen [38] have investigated the relationship between mindfulness and supportive communication process and suggested that mindfulness provides more compassionate and beneficial emotional support within communications with others and to improve the personal relationships. As for the part of concentration, Fountain-Zaragoza, et al. [39] showed that dispositional mindfulness was negatively associated with task-unrelated thought, reflecting that the athletes with higher mindfulness likely have better concentration to focus on task-related cues and avoid making mistakes by task-irrelated interferences in the competition (e.g., audience noise or poor weather). Furthermore, similar to our findings, Walker [14] observed that dispositional mindfulness is positively related to self-confidence, which is the foremost goal of sports psychology [23] and is considered as a key factor in an athlete’s return to sports after an injury [40]. The increase in self-confidence therefore suggests that athletes having a stable level of self-confidence is extremely important for overcoming challenges [14]. Lastly is the linkage between mindfulness and peaking under pressure and coping with adversity. While athletes will regard the stress as a threat when feeling uncontrollable stress that impairs their sports performance or even reduces enthusiasm and causes burnout [41], mindfulness may help the athletes to cope with stress, and reduce their reaction to stress in order to improve their sports performance [9].

Except for another cross-sectional study that observed the relationship between mindfulness and psychological skill, this study has also examined the alterations of mindfulness and psychological skills following a mindfulness-based intervention [9,42,43,44]. Vidic, St. Martin, and Oxhandler [42] found that ACSI-28 overall scores were significantly increased among NCAA women basketball athletes following a 10-session mindfulness training, and a similarly significant improvement was observed in concentration, self-confidence/achievement motivation, goal setting/mental preparation, coping with adversity, peaking under pressure, and a decrease in perceived stress following mindfulness training. In addition, the qualitative results also found that mindfulness training increases awareness of the ability of mindfulness. Jones, Kaur, Miller, and Spencer [43] also adopted ACSI-28 to explore the effects of the eight-week mindfulness training for rowers, and they observed that the rowers improved their mindfulness level, exercise coping ability, and athletic performance. Similar results were achieved for handball athletes, who after an eight-week mindfulness intervention improved their coping with adversity, coachability, concentration, goal-setting, mental preparation, and freedom from worry [44]. The study also suggested that mindfulness-based interventions benefit the mindfulness level and stress management among athletes by reducing their physiological stress response [9]. These studies could provide the evidence for athletes with a lower dispositional mindfulness to enhance their mindfulness level via a mindfulness-based intervention and later to positively link it to their psychological skills.

The finding of a significant positive correlation between dispositional mindfulness and overall, positive effort, and the anti-pressure of mental toughness, is partially consistent with previous studies. Walker [14] recruited 484 adolescent female hockey players and examined their association of mindfulness and mental toughness, by using the Child and Adolescent Mindfulness Measure (CAMM) and the Sport Mental Toughness Questionnaire (SMTQ). They found that a significant positive correlation existed between dispositional mindfulness and comprehensive mental toughness. Similar results were also observed by Abdul Rafeeque and Sultana [45], who targeted 323 track and field athletes. Measuring dispositional mindfulness and mental toughness by using the Mindfulness Mindlessness Scale (MMS), and the Mental Toughness Scale (MTS), they observed that mindfulness is positively related to mental toughness.

Our study examines the mental toughness measure designed by Huang [35] in which three domains were included: positive effort, anti-pressure, and endurance. Positive effort is believed to reflect striving to progress and never giving up in a difficult training environment. Our study provided preliminary evidence of the association between dispositional mindfulness and effort. In addition, the results of anti-pressure in mental toughness are similar to peaking under pressure and coping with adversity in APSI, which also had a result similar to results of past studies that there is a positive correlation between mindfulness and less stress [17,42]. It is notable that we observed a non-significant correlation between mindfulness and endurance of mental toughness, which is contrary to other studies that have demonstrated a negative association between dispositional mindfulness and pain response [46]. Jones and Parker [15] showed that mindfulness partially mediates the relationship between mental toughness and pain catastrophizing. This result indicates that the mentally tough athletes with higher dispositional mindfulness can reduce fear of pain [15]. Furthermore, there are studies that demonstrate that mindfulness-based interventions increased the pain tolerance for those athletes who were injured [47]. Specifically, we speculate that there are two possibilities. First, due to the questionnaire used in this study being different from previous studies, the concepts of the questions may not be the same. Second, in this study, endurance represents a response to poor health or an unbearable body condition [35]. However, modern athletes have more knowledge about sport injuries, so they do not have to be forced to endure the pain of their body and avoid, as much as possible, being injured.

In terms of the underlying mechanism between mindfulness, psychological skills, and mental toughness, the many domains contained in APSI and TMTIS are considered as an underlying mechanism of mindfulness influencing sports performance. For example, Birrer, Röthlin and Morgan [4] have pointed out that those athletes with higher dispositional mindfulness may improve their levels of performance-relevant psychological outcomes through several impact mechanisms, such as attention, emotional regulation, and values clarifications. Concentration is one of the attention categories [48], implying that the more mindful athletes should pay attention to placing it on their task-related goals list [4]. Whereas, applying coping skills to relieve stress may also be related to emotional regulation [13]. Moreover, Birrer et al. [4] also suggested that how athletes clarify their values will mediate the relationship between mindfulness and motivation. Overall, the current study provides initial evidence that there is an advantageous relationship between dispositional mindfulness and psychological skills and mental toughness in multiple-sport populations. This may indirectly mean that dispositional mindfulness will help individuals to have better performance-relevant psychological outcomes through engaging some specific mechanisms, and that this will contribute positively to their sports performance.

The results must be interpreted carefully in terms of related methodological limitations. Although our study recruited athletes from a variety of sports (i.e., taekwondo, judo, archery, wushu, and tennis) as a statistical strength, the sample is still considered relatively small. A larger sample could have increased the statistical power of the parameter estimates. In order to deepen the understanding of the association between dispositional mindfulness, psychological skills, and the mental toughness of athletes, future studies need to recruit similar diverse athletes with larger group samples. Another weakness of this study is the use of the cross-sectional design, which may limit the inference of causal relationships between these psychological characteristics. Future research should adopt longitudinal studies or intervention studies to examine causal relationships and whether mindfulness-based interventions could improve the changes in dispositional mindfulness, psychological skills, and mental toughness. Third, only run the correlation analysis to investigate the correlation between variables in this study. However, this work is a preliminary study to examine the relationship between mindfulness, mental skills and mental toughness in multiple-sport populations. In the next step, future studies could also adopt a prediction model to explore possible pathways of causality between mindfulness, psychological skills and mental toughness. Lastly, the present study only considered the relationship between dispositional mindfulness, psychological skills, and mental toughness, but there are many psychological characteristics that would affect the athletes and their performance, such as anxiety, flow experience, and perfectionism. Consequently, it is also necessary to investigate the relationship between these three and other performance-related psychological characteristics.

## 5. Conclusions

The present study shows that dispositional mindfulness is positively related to comprehensive psychological skills, as well as mental toughness, which includes the TMTIS overall score, positive effort, and anti-pressure. The positive linkage among dispositional mindfulness, psychological skills, and mental toughness among multiple-sport athletes suggests that it is perhaps appropriate to further develop dispositional mindfulness in athletes and mindfulness-based interventions might be considered in the future.

## Figures and Tables

**Table 1:** Demographic characteristics and level of psychological characteristics.

Variables	*M* ± *SD*
Demographic characteristics	
Sample size (*n*)	101
Gender (M/F)	72/29
Age (years)	20.70 ± 1.31
Educational level (years)	14.99 ± 1.03
Sport experiences (years)	9.58 ± 3.47
Level of psychological characteristics	
CMAAS	4.34 ± 0.61
APSI overall	3.37 ± 0.50
Motivation	3.20 ± 0.81
Coachability	4.01 ± 0.68
Concentration	3.52 ± 0.78
Confidence	2.94 ± 0.75
Peaking and coping	3.20 ± 0.69
TMTIS overall	3.85 ± 0.53
Positive effort	3.98 ± 0.67
Anti-pressure	3.41 ± 0.64
Endurance	4.17 ± 0.59

Note. CMAAS = Chinese Version Mindful Attention Awareness Scale; APSI = Athletic Psychological Skills Inventory; TMTIS = Trait Mental Toughness Inventory for Sport; Peaking and coping = Peaking under pressure and coping with adversity; *M* = Mean; *SD* = Standard deviation.

**Table 2:** Pearson correlation coefficients for CMAAS and APSI.

	1	2	3	4	5	6	7
1. CMAAS	-	0.36 **	0.24 *	0.23 *	0.21 *	0.32 **	0.21 *
2. APSI overall		-	0.73 **	0.52 **	0.61 **	0.70 **	0.81 **
3. Motivation			-	0.41 **	0.13	0.43 **	0.46 **
4. Coachability				-	0.14	0.05	0.20 *
5.Concentration					-	0.27 **	0.48 **
6. Confidence						-	0.60 **
7. Peaking and coping							-

Note. CMAAS = Chinese Version Mindful Attention Awareness Scale; APSI = Athletic Psychological Skills Inventory; Peaking and coping = Peaking under pressure and coping with adversity; * = *p* < 0.05; ** = *p* < 0.01.

**Table 3:** Pearson correlation coefficients for CMAAS and TMTIS.

	1	2	3	4	5
1. CMAAS	-	0.27 **	0.26 **	0.30 **	0.12
2. TMTIS overall		-	0.89 **	0.80 **	0.80 **
3. Positive effort			-	0.57 **	0.63 **
4. Antipressure				-	0.40 **
5. Endurance					-

Note. CMAAS = Chinese Version Mindful Attention Awareness Scale; TMTIS = Trait Mental Toughness Inventory for Sport; ** = *p* < 0.01.

## Data Availability

All datasets generated for this research are included in this published article.

## References

[1] Gooding A., Gardner F.L. (2009). An investigation of the relationship between mindfulness, preshot routine, and basketball free throw percentage. J. Clin. Sport Psychol..

[2] Jones M.I., Parker J.K. (2015). A conditional process model of the effect of mindfulness on 800-m personal best times through pain catastrophising. J. Sports Sci..

[3] Gardner F.L., Moore Z.E. (2020). Mindfulness in sport contexts. Handbook of Sport Psychology.

[4] Birrer D., Röthlin P., Morgan G. (2012). Mindfulness to enhance athletic performance: Theoretical considerations and possible impact mechanisms. Mindfulness.

[5] Joshi V.A., Kalode R.D. (2019). A comparative study of mental toughness in elite taekwondo players. Int. J. Physiol. Nutr. Physic. Educ..

[6] Kabat-Zinn J. (2003). Mindfulness-based interventions in context: Past, present, and future. Clin. Psychol..

[7] White L. (2014). Mindfulness in nursing: An evolutionary concept analysis. J. Adv. Nurs..

[8] Medvedev O.N., Krägeloh C.U., Narayanan A., Siegert R.J. (2017). Measuring mindfulness: Applying generalizability theory to distinguish between state and trait. Mindfulness.

[9] Mehrsafar A.H., Strahler J., Gazerani P., Khabiri M., Sanchez J.C.J., Moosakhani A., Zadeh A.M. (2019). The effects of mindfulness training on competition-induced anxiety and salivary stress markers in elite Wushu athletes: A pilot study. Physiol. Behav..

[10] Nien J.T., Wu C.H., Yang K.T., Cho Y.M., Chu C.H., Chang Y.K., Zhou C. (2020). Mindfulness training enhances endurance performance and executive functions in athletes: An event-related potential study. Neural. Plast..

[11] Röthlin P., Horvath S., Trösch S., Holtforth M., Birrer D. (2020). Differential and shared effects of psychological skills training and mindfulness training on performance-relevant psychological factors in sport: A randomized controlled trial. BMC Psychol..

[12] Kee Y.H., Wang C.K.J. (2008). Relationships between mindfulness, flow dispositions and mental skills adoption: A cluster analytic approach. Psychol. Sport Exerc..

[13] Josefsson T., Ivarsson A., Lindwall M., Gustafsson H., Stenling A., Böröy J., Mattsson E., Carnebratt J., Sevholt S., Falkevik E. (2017). Mindfulness mechanisms in sports: Mediating effects of rumination and emotion regulation on sport-specific coping. Mindfulness.

[14] Walker S.P. (2016). Mindfulness and mental toughness among provincial adolescent female hockey players. S. Afr. J. Sports Med..

[15] Jones M.I., Parker J.K. (2018). Mindfulness mediates the relationship between mental toughness and pain catastrophizing in cyclists. Eur. J. Sport Sci..

[16] Lee Y.H. (2020). The role of mindfulness and occupational stress in the goal orientations of development and winning. Sport Manag. Rev..

[17] Gustafsson H., Skoog T., Davis P., Kenttä G., Haberl P. (2015). Mindfulness and its relationship with perceived stress, affect, and burnout in elite junior athletes. J. Clin. Sport Psychol..

[18] Li C., Zhu Y., Zhang M., Gustafsson H., Chen T. (2019). Mindfulness and athlete burnout: A systematic review and meta-analysis. Int. J. Environ. Health Res..

[19] Blijlevens S.J.E., Elferink-Gemser M.T., Wylleman P., Bool K., Visscher C. (2018). Psychological characteristics and skills of top-level Dutch gymnasts in the initiation, development and mastery stages of the athletic career. Psychol. Sport Exerc..

[20] Birrer D., Morgan G. (2010). Psychological skills training as a way to enhance an athlete’s performance in high-intensity sports. Scand. J. Med. Sci. Sports.

[21] Otten M. (2009). Choking vs. clutch performance: A study of sport performance under pressure. J. Sport Exerc. Psychol..

[22] Ong N.C.H. (2019). Assessing objective achievement motivation in elite athletes: A comparison according to gender, sport type, and competitive level. Int. J. Sport Exerc. Psychol..

[23] Beaumont C., Maynard I.W., Butt J. (2015). Effective ways to develop and maintain robust sport-confidence: Strategies advocated by sport psychology consultants. J. Appl. Sport Psychol..

[24] Sanchez-Lopez J., Silva-Pereyra J., Fernandez T. (2016). Sustained attention in skilled and novice martial arts athletes: A study of event-related potentials and current sources. Peer J..

[25] Ponnusamy V., Lines R.L.J., Zhang C.Q., Gucciardi D.F. (2018). Latent profiles of elite Malaysian athletes’ use of psychological skills and techniques and relations with mental toughness. Peer J..

[26] Kristjánsdóttir H., Erlingsdóttir A.V., Sveinsson G., Saavedra J.M. (2018). Psychological skills, mental toughness and anxiety in elite handball players. Pers. Individ. Dif..

[27] Gucciardi D.F. (2016). Mental toughness as a moderator of the intention–behaviour gap in the rehabilitation of knee pain. J. Sci. Med. Sport.

[28] Gordon S., Anthony D.R., Gucciardi D.F. (2017). A case study of strengths-based coaching of mental toughness in cricket. Int. J. Sport Psychol..

[29] Lin Y., Clough P.J., Welch J., Papageorgiou K.A. (2017). Individual differences in mental toughness associate with academic performance and income. Pers. Individ. Dif..

[30] Moen F., Federici R.A., Abrahamsen F. (2015). Examining possible relationships between mindfulness, stress, school-and sport performances and athlete burnout. Int. J. Sports Sci. Coach.

[31] Chang J.H., Lin Y.C., Huang C.L. (2011). Psychometric properties of the chinese translation of Mindful Attention Awareness Scale (CMAAS). Psychol. Test.

[32] Brown K.W., Ryan R.M. (2003). The benefits of being present: Mindfulness and its role in psychological well-being. J. Pers. Soc. Psychol..

[33] Chiou Y.H., Chi L.K. (2001). The development of the athletic psychoogical skills inventory. Bull. Sport Exerc. Psychol. Taiwan.

[34] Smith R.E., Schutz R.W., Smoll F.L., Ptacek J.T. (1995). Development and validation of a multidimensional measure of sport-specific psychological skills: The athletic coping skills Inventory-28. J. Sport Exerc. Psychol..

[35] Huang C.J. (2003). Establishing the Construct of Mental Toughness for Sport: Preliminary Investigation and Instrument Development.

[36] Ruffault A., Bernier M., Juge N., Fournier J.F. (2016). Mindfulness may moderate the relationship between intrinsic motivation and physical activity: A cross-sectional study. Mindfulness.

[37] Wang M.Y., Huang R.Y., Liao C.C., Nai H.F. (2015). Study on the correlation of the leading way of table-tennis coach on the sport psychological skills of athletes. Leis. Ind. Res..

[38] Jones S.M., Hansen W. (2015). The impact of mindfulness on supportive communication skills: Three exploratory studies. Mindfulness.

[39] Fountain-Zaragoza S., Londerée A., Whitmoyer P., Prakash R.S. (2016). Dispositional mindfulness and the wandering mind: Implications for attentional control in older adults. Conscious. Cogn..

[40] Podlog L., Banham S.M., Wadey R., Hannon J.C. (2015). Psychological readiness to return to competitive sport following injury: A qualitative study. Sport Psychol..

[41] Bali A. (2015). Psychological factors affecting sports performance. Int. J. Phys. Educ. Sports Health.

[42] Vidic Z., Martin M.S., Oxhandler R. (2017). Mindfulness intervention with a U.S. women’s NCAA division I basketball team: Impact on stress, athletic coping skills and perceptions of intervention. Sport Psychol..

[43] Jones B.J., Kaur S., Miller M., Spencer R. (2020). Mindfulness-based stress reduction benefits psychological well-being, sleep quality, and athletic performance in female collegiate rowers. Front. Psychol..

[44] Terzioğlu Z.A., Yildiz M., Çakir S.G. (2020). Examining the effectiveness of mindfulness based training program on female handball players’ psychological skills and coping with stress strategies. Turk. J. Sport Exerc..

[45] Abdul Rafeeque T.C., Sultana D. (2016). Mediating role of mindfulness on the relationship between mental toughness and athletics performance of inter university track and field athletes. Int. J. Phys. Educ. Sports Health.

[46] McCracken L.M., Gauntlett-Gilbert J., Vowles K.E. (2007). The role of mindfulness in a contextual cognitive-behavioral analysis of chronic pain-related suffering and disability. Pain.

[47] Mohammed W.A., Pappous A., Sharma D. (2018). Effect of mindfulness based stress reduction (MBSR) in increasing pain tolerance and improving the mental health of injured athletes. Front. Psychol..

[48] Buehner M., Krumm S., Ziegler M., Pluecken T. (2006). Cognitive abilities and their interplay: Reasoning, crystallized intelligence, working memory components, and sustained attention. J. Individ. Differ..

